# The erector spinae block: a novel approach to pain management in acute appendicitis

**DOI:** 10.1186/s13089-022-00281-7

**Published:** 2022-07-26

**Authors:** Jonathan Brewer, Holly Conger, Robert Rash

**Affiliations:** Department of Emergency Medicine, UT Southwestern Medical Center, 5323 Harry Hines Boulevard, E4.300, Dallas, TX 75390-8579 USA

**Keywords:** Emergency medicine, POCUS, Ultrasound, Regional anesthesia, Pain management, Erector spinae plane block, Appendicitis, Analgesia, Acute abdominal pain, Multimodal pain control, Emergency department

## Abstract

**Background:**

Acute abdominal pain is one of the most common complaints that patients present with in the emergency room and has long been a challenge to effectively manage without relying on opioid analgesia. The use of ultrasound-guided peripheral nerve blocks (UGRA) represents a new frontier in multimodal pain control regimens in the acute setting. An erector spinae plane (ESP) block is believed to mediate pain relief in multiple dermatomes through blockage of both visceral and somatic nerves. Analgesia provided by a single injection can help keep a patient comfortable for hours without breakthrough pain and the subsequent need for frequent redosing of opioid pain medication. To this date, there is very limited evidence of an ESP block in the utilization of acute appendicitis in the emergency department.

**Case report:**

This case report presents a 26-year-old female with a past medical history of polycystic ovarian syndrome (PCOS) and a tubal ligation that presented with 7/10 right lower quadrant abdominal pain that began 1 h prior to arrival. She stated that she felt like this was similar to her PCOS exacerbations in the past. During her evaluation, she underwent a computed tomography (CT) scan of her abdomen and pelvis that was remarkable for acute, uncomplicated appendicitis. She was given 4 mg of morphine for her pain with little response, so the offer was made for an erector spinae block that the patient elected to receive. After being consented both for the procedure and for research, she received a right-sided erector spinae block with 20 mL’s of 0.2% ropivacaine (2 mg/mL) at the L1 vertebral level. After approximately 15 min, she stated that she had a reduction in her pain from a 6/10 to a 1/10 that persisted throughout the rest of her stay in the emergency department.

## Background

Appendicitis is one of the most common causes of acute abdominal pain in both adults and children with nearly 300,000 appendectomies performed each year. Right lower quadrant (RLQ) or periumbilical abdominal pain were the most common presenting symptoms in patients with acute appendicitis [1]. Antibiotic therapy and analgesia are mainstays of appendicitis treatment in addition to surgical intervention. Diagnosis of appendicitis, and then surgical intervention, may take hours in the emergency department during which a patient may suffer from inadequately treated pain [2]. Visceral pain is traditionally more difficult to manage than somatic pain with conventional pain medications and is often not well controlled with the use of opioids or anti-inflammatory medications [3]. In this case report, we describe how an ESP block can be useful in providing analgesia in the setting of acute appendicitis.

## Case presentation

**Fig. 1:**
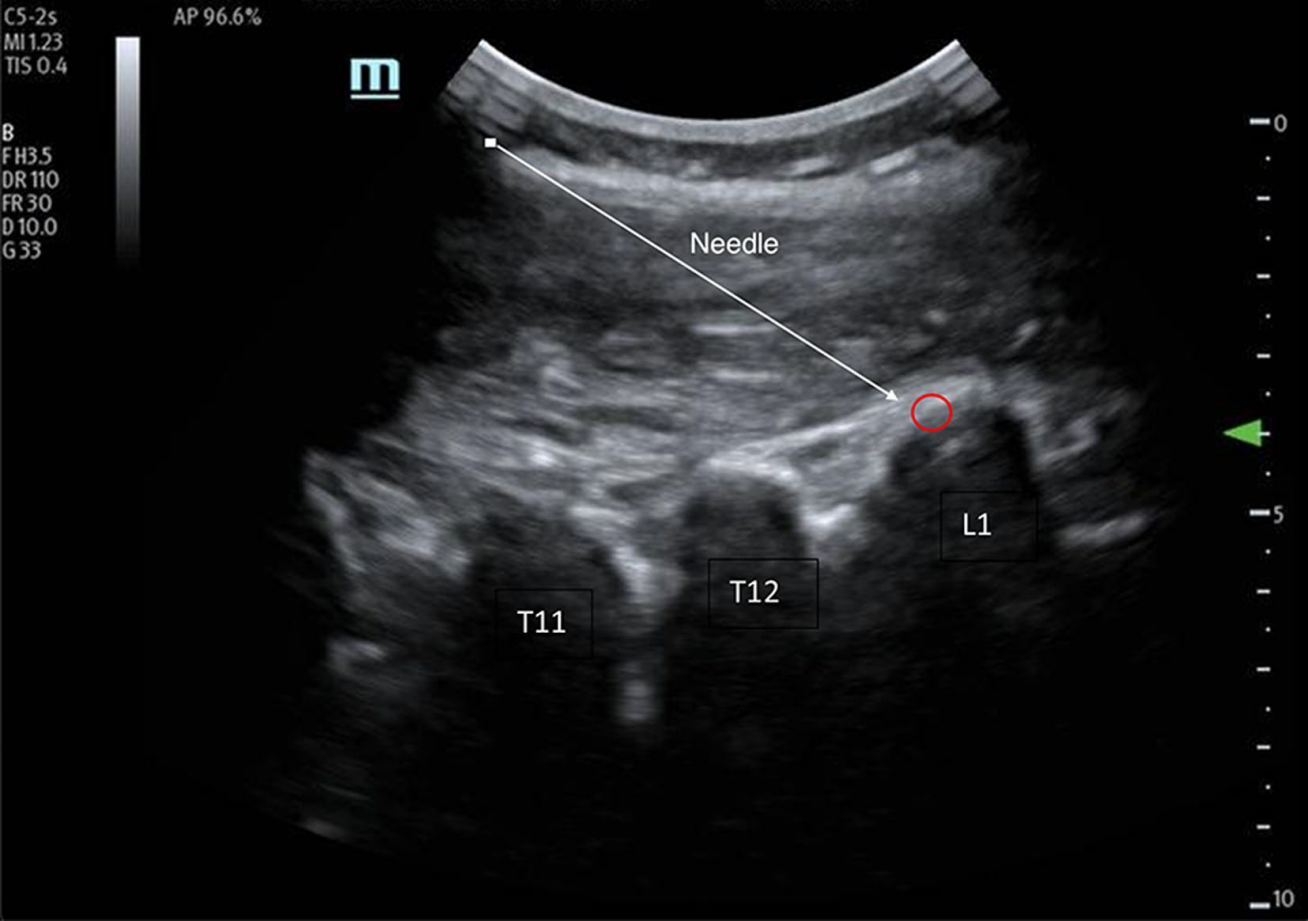
Longitudinal needle guidance to the L1 transverse process

This is the case of a 26-year-old G3P2A1 female with a past medical history of polycystic ovarian syndrome (PCOS) status-post tubal ligation that presented with right lower quadrant pain that began 1 h prior to arrival. Stated that this had an acute onset and described it as a constant, stabbing pain that was worst when she moved. She stated that this felt like her past PCOS exacerbations and denied any other infectious symptoms or recent trauma. Her vital signs upon arrival were remarkable for a mild hypotension to 93/48 and tachycardia to 102, but she was afebrile and these both improved with administration of 1L of lactated ringers. On exam, she was found to have tenderness in her right lower quadrant with negative Rovsing and obturator signs. Her labs were grossly unremarkable with no leukocytosis and no electrolyte abnormalities. Her urine pregnancy test was also found to be negative, so the decision was made to pursue a computed tomography (CT) scan of the abdomen and pelvis with intravenous contrast instead of a pelvic ultrasound. By this point, her pain and nausea were beginning to increase so 4 mg of morphine was ordered along with 4 mg of ondansetron for her nausea. She underwent the CT scan which was remarkable for a mild, uncomplicated acute appendicitis without perforation or abscess formation. Surgery was consulted and the patient was consented for an appendectomy. At this time, the patient was requesting more pain medication as she felt that her pain was continuing to increase. After a discussion regarding risks and benefits of more opioid medication versus an erector spinae block, the patient elected to pursue an erector spinae block. The patient was consented for both a block and for research purposes and the procedure was prepared. The patient was situated in a sitting position, similar to a lumbar puncture, with her lumbar vertebrae exposed. While many patients are positioned in the prone position with the operator at the head of the bed facing caudally, our patient’s abdominal pain was exacerbated with the prone position so the decision was made to place her in a less ideal sitting position. One should note that when a patient is placed upright, this may lead to bradycardia or hypotension during the procedure. The operator was positioned behind her and the patient was prepped and draped. Using sterile technique and ultrasound guidance with a curvilinear probe (due to patient’s body habitus), the L1 vertebra was located in the parasagittal plane. The probe was then moved right approximately 2 cm lateral to the spinous process (Fig. 1) and a 21 g Pajunk single-shot 100-mm needle was inserted in a cranial-to-caudal distribution until contact was made with the posterior surface of the transverse process. Hydro-dissection with 10 mL of normal saline with direct visualization confirmed needle tip placement in the fascial plane. After negative aspiration, 20 mL of 0.2% ropivacaine (2 mg/mL) were injected in aliquots of 4–5 mL with repeat aspiration in between. The patient remained on the cardiac monitor for the duration of the block and was monitored for any signs and symptoms of local anesthetic systemic toxicity. Over the next 15 min, she was found to have a significant decrease in pain from a 6/10 to a 1/10 and did not require any more pain medication throughout her stay in the emergency department and initial stay on the floor. Surgery elected to provide an as needed pain regimen of acetaminophen with codeine (300 mg with 30 mg), ketorolac (15 mg), and methocarbamol (500 mg). The patient did not request her first dose of pain medication until 6 h after the block had been performed and required minimal repeat non-opioid doses throughout the night. She underwent an appendectomy the following morning and was discharged afterwards without any complications.

## Discussion

This is a case of a 26-year-old female presenting with RLQ abdominal pain. She was found to have acute appendicitis without perforation in the emergency department. There are case reports and literature discussing the use of US-guided ESP blocks for abdominal pain in the perioperative setting, but little about the use of such a block in the setting of acute abdominal pain in the emergency room [4]. Pain from acute appendicitis is primarily mediated through visceral pain receptors, but also involves somatic receptors of the abdominal wall that become irritated as the peritoneum becomes inflamed from a developing infection. Afferent visceral pain fibers that transmit signals distention, ischemia, inflammation, and other noxious stimuli back to the spinal cord are composed of C fibers that originate within the walls of abdominal visceral organs [5]. These nerve fibers travel with the sympathetic nerves as they transverse back to the spinal cord. These nerves run together as the splanchnic nerves before branching off to run through the dorsal root ganglia, and then finally enter the spinal cord via the rami-communicantes bridging to the spinal nerves [6]. Somatic afferent fibers, in contrast, run through peripheral nerves from the dermatomes they innervate and back to the dorsal horn of the spinal cord through the ventral rami of the spinal nerves [7]. In order for pain to be adequately controlled, the analgesia must affect both aforementioned pain pathways. An ESP block involves injection of anesthetic into the erector spinae muscles that then diffuses through the deep fascial plane and into the paravertebral space, where it contacts the dorsal and ventral rami of spinal nerves, but also the rami-communicantes carrying sympathetic nerve fibers. The hypothesis for this mechanism is based on cadaveric studies in which the injection hydrodissects the tissue plane and spreads craniocaudally through across multiple spinal levels to encompass the majority of fibers innervating one area of the body [8]. This provides analgesia by blocking both the visceral and somatic fibers innervating the spinal levels providing innervation [9]. In our case, this injection resulted in significant pain reduction for the patient. In addition, the ESP block provided pain relief for the remainder of this patient’s ED stay, and she did not require any further pain medications while in the ED. The block in conjunction with minimal pain medications throughout the night kept the patient comfortable until her surgery the following morning.

## Conclusion

In conclusion, incorporating an ESP block into the multimodal pain regimen for acute abdominal pain in the ED appears to be a viable option for both visceral and somatic pain. Abdominal pain is a common complaint that other classes of medication are often inadequate at effectively controlling. In our case, the block successfully treated visceral and somatic pain and lessened the need for additional opioid medication while the patient awaited surgical intervention. The use of US-guided peripheral nerve blocks for treating abdominal pain in the acute setting is an area of study that should be further explored.

## Data Availability

All data, figures, and materials are of original work.
